# Genetic Variants of Wnt Transcription Factor *TCF-4* (*TCF7L2*) Putative Promoter Region Are Associated with Small Intestinal Crohn's Disease

**DOI:** 10.1371/journal.pone.0004496

**Published:** 2009-02-16

**Authors:** Maureen J. Koslowski, Irmgard Kübler, Mathias Chamaillard, Elke Schaeffeler, Walter Reinisch, Guoxing Wang, Julia Beisner, Alexander Teml, Laurent Peyrin-Biroulet, Stefan Winter, Klaus R. Herrlinger, Paul Rutgeerts, Séverine Vermeire, Rachel Cooney, Klaus Fellermann, Derek Jewell, Charles L. Bevins, Matthias Schwab, Eduard F. Stange, Jan Wehkamp

**Affiliations:** 1 Dr. Margarete Fischer-Bosch-Institute of Clinical Pharmacology, University of Tübingen, Stuttgart, Germany; 2 Inserm, U801, Institut Pasteure Lille, Université Lille 2, Lille, France; 3 Department of Internal Medicine I, Robert Bosch Hospital, Stuttgart, Germany; 4 Department of Internal Medicine IV, Medical University of Vienna, Vienna, Austria; 5 Service d'Hépato-gastro-entérologie, CHU Nancy-Brabois allée du Morvan, Vandceuvre-lès-Nancy, France; 6 Division of Mathematics, Institute of Stochastics and Applications, University of Stuttgart, Stuttgart, Germany; 7 Division of Gastroenterology, University of Leuven Hospitals, Leuven, Belgium; 8 Medical Science Division, John Radcliffe Hospital, Oxford, Oxford, United Kingdom; 9 Department of Microbiology and Immunology, School of Medicine, University of California Davis, Davis, California, United States of America; 10 Department of Clinical Pharmacology, University of Tübingen, Tübingen, Germany; Ohio State University Medical Center, United States of America

## Abstract

Reduced expression of Paneth cell antimicrobial α-defensins, human defensin (HD)-5 and -6, characterizes Crohn's disease (CD) of the ileum. TCF-4 (also named TCF7L2), a Wnt signalling pathway transcription factor, orchestrates Paneth cell differentiation, directly regulates the expression of HD-5 and -6, and was previously associated with the decrease of these antimicrobial peptides in a subset of ileal CD. To investigate a potential genetic association of *TCF-4* with ileal CD, we sequenced 2.1 kb of the 5′ flanking region of *TCF-4* in a small group of ileal CD patients and controls (n = 10 each). We identified eight single nucleotide polymorphisms (SNPs), of which three (rs3814570, rs10885394, rs10885395) were in linkage disequilibrium and found more frequently in patients; one (rs3814570) was thereby located in a predicted regulatory region. We carried out high-throughput analysis of this SNP in three cohorts of inflammatory bowel disease (IBD) patients and controls. Overall 1399 healthy individuals, 785 ulcerative colitis (UC) patients, 225 CD patients with colonic disease only and 784 CD patients with ileal involvement were used to determine frequency distributions. We found an association of rs3814570 with ileal CD but neither with colonic CD or UC, in a combined analysis (allele positivity: OR 1.27, 95% CI 1.07 to 1.52, p = 0.00737), which was the strongest in ileal CD patients with stricturing behaviour (allele frequency: OR 1.32, 95% CI 1.08 to1.62, p = 0.00686) or an additional involvement of the upper GIT (allele frequency: OR 1.38, 95% CI 1.03 to1.84, p = 0.02882). The newly identified genetic association of *TCF-4* with ileal CD provides evidence that the decrease in Paneth cell α-defensins is a primary factor in disease pathogenesis.

## Introduction

Inflammatory bowel disease (IBD), a chronic inflammation of the intestine, is commonly classified into ulcerative colitis (UC) and Crohn's disease (CD) on the basis of clinical features and histopathology [1]. Whereas UC is typically restricted to the colon, CD can occur at many sites, predominantly in the small intestinal ileum, the colon, or in both locations. Emerging details of disease pathogenesis support the current concept that ongoing immune activation in IBD is driven by bacterial microbiota, possibly as a result to an attenuated antimicrobial barrier in genetically predisposed individuals [1]–[3]. Both UC and CD have a complex polygenic, multifactorial background, with a coincidence of susceptibility genes and environmental factors involved in pathogenesis. It is likely that different genetically affected factors may explain the various clinical patterns of IBD, especially location of disease in CD, which is stable over time [4]–[6]. Different explanations for disease location, including a central role of small intestinal Paneth cells and other defects in intestinal innate immunity, were the focus of recent discussion [2]. For ileal CD, reduced expression of small intestinal Paneth cell α-defensins HD-5 and -6 (DEFA5 and DEFA6) has been described in several cohorts [7]–[12]. The defensin deficiency is proposed to attenuate the antibacterial host defense capacity of the intestinal mucosa, and may initiate and/or perpetuate the chronic inflammation characterizing the disease at this site [7]–[12]. We recently reported one mechanism to explain, in part, the decrease of these antimicrobial peptides [9], [13]: A reduced expression of the Wnt pathway transcription factor TCF-4 (also known as transcription factor 7-like 2), which directly controls Paneth cell defensin expression (HD-5, HD-6, and orthologous mouse cryptdin peptides [9], [13]).

Wnt proteins are a family of secreted morphogenes that play an important role in regulating cell fate and differentiation during embryogenesis [14]. The Wnt signalling pathway is induced by binding of Wnt family proteins to cell surface receptors, leading to stabilization of cytoplasmatic β-catenin, translocation of this regulatory protein into the nucleus, formation of a complex with transcription factors of the Tcf/Lef family and subsequently the activation of various target genes [13]. In the small intestine, epithelial cells transit through differentiation steps initiated in progenitor cells, which reside adjacent to Paneth cells at the base of the crypts [15]. Wnt signalling helps to maintain an undifferentiated state of the intestinal stem cells [16], [17] and, paradoxically, also regulates positioning, differentiation and maturation of Paneth cells [13], [18]. The Paneth cell gene program is critically dependent on TCF-4 [13]. Using a rodent model, we observed that very small changes (a 50% decrease of TCF-4 levels) are sufficient to compromise mouse Paneth cell cryptidin expression as well as its corresponding antimicrobial function against several bacterial species. We also reported that a reduced level of TCF-4 expression and activity was associated with a decrease of Paneth cell α-defensin levels in CD of the small intestine. The decrease of TCF-4 expression was found to be independent of inflammation in the tissue specimens, and also independent of the 1007fsinsC SNP in *NOD2*, a mutation in this pattern recognition receptor which has previously been associated with ileal CD [9]. We hypothesized that decreased TCF-4 expression might be the result of primary genetic variances in *TCF-4*, at least in some patients with ileal CD. Since there was a decrease in *TCF-4* mRNA levels in these studies, an aberration in the promoter region of *TCF-4* could be a possible explanation. Thus, the aim of this study was to sequence the promoter region of the *TCF-4* gene in a group of patients with ileal CD to identify potential polymorphisms and to perform a subsequent association study on candidate genetic variants in well-defined cohorts of patients. We identified a total of 8 SNP variants, of which three (rs3814570, rs10885394, rs10885395) were in linkage disequilibrium and seemed to exhibit a higher frequency in ileal CD patients. One of these SNPs was found to be located in a putative regulatory region. We carried out high- throughput analysis of this SNP in three IBD cohorts from Oxford, Leuven and Vienna [19]–[21]. Herein we report an association of the SNP rs3814570 with ileal involvement of CD, but not with colonic CD or UC.

## Methods

### Patients and human material

For genetic analysis, we obtained DNA samples from a patient cohort of Caucasians with Crohn's disease (N = 259) or ulcerative colitis (N = 149) from the University Hospital in Vienna, as well as a control group of unrelated, healthy Caucasian blood donors in Stuttgart (N = 833). For subsequent testing, we obtained DNA samples from Caucasians with Crohn's disease (N = 277), UC (N = 74) and healthy controls (N = 242) from the University of Leuven, Belgium (*3*) as well as an additional third Caucasian cohort from Oxford with DNA of Caucasian healthy individuals (N = 324), UC (N = 562) and CD (N = 473) patients. In line with the Montreal classification (*4*) three subgroups were defined: ileal disease only (L1), colonic disease only (L2) and ileo colonic disease (L3). A total of 1399 randomly recruited healthy control individuals, 785 UC patients, 225 CD (L2) patients with disease limited to the colon and 784 CD patients with ileal involvement (L1+L3) were used to elucidate the frequency distribution of SNPs [19]–[21]. The numbers of patient subgroups and controls in the different cohorts are shown in Table 1 and detailed statistical analyses are provided in Table 2. To exclude major differences between the groups in age or gender, CD patients as well as controls were sub grouped according to these criteria (Table 3). Additional points of interest were the behaviour as well as the aggressiveness of the disease. We therefore decided to separately test for an association with inflammatory, stricturing and penetrating behaviour as well as an association of the variant with surgery for Crohn's disease. Finally we checked patients with an additional involvement of the upper gastrointestinal tract (L4). The study was approved by the ethics committees of the Medical University Vienna, Austria, the University Hospital Tübingen, Germany, the University of Leuven, Belgium and the Oxford Radcliffe Hospital Trust. All patients gave informed and written consent for their DNA to be analyzed for this study.

**Table 1:** Overview of the origin of samples from IBD patients and healthy controls.

	Controls	UC	CD (L1)	CD (L2)	CD (L3)
Vienna	833*	149	54	55	150
Leuven	242	74	81	45	151
Oxford	324	562	94	125	254

* healthy blood donors from Stuttgart.

**Table 2:** *TCF-4* (*TCF7L2*) rs3814570 frequency distribution and statistical analysis of combined cohort samples.

All												
	controls	UC	CD (L1)	CD (L3)	CD (L1+L3)	CD (L2)	CD	IBD	controls		controls	
	n(%)	n(%)	n(%)	n(%)	n(%)	n(%)	n(%)	n(%)	<> CD		<> UC	
									**C<>T**	**CC<>CT+TT**	**C<>T**	**CC<>CT+TT**
rs3814570	1399 (100%)	785 (100%)	229 (100%)	555 (100%)	784 (100%)	225 (100%)	1009 (100%)	1794 (100%)	1.16; p = 0.02246	1.18; p = 0.04358	0.98; p = 0.78946	1.01; p = 0.94441
									**Armitage's trend**		**Armitage's trend**	
C/C	797 (56,97%)	446 (56,82%)	113 (49,34%)	287 (51,71%)	400 (51,02%)	133 (59,11%)	533 (52,82)	979 (54,57%)	1.15; p = 0.02852		0.97; p = 0.79641	
C/T	488 (34,88%)	282 (35,92%)	94 (41,05%)	209 (37,66%)	303 (38,65%)	73 (32,44%)	376 (37,27%)	658 (36,68%)	**controls**		**controls**	
T/T	114 (8,15%)	57 (7,26%)	22 (9,6%)	59 (10,63%)	81 (10,33%)	19 (8,44%)	100 (9,91%)	157 (8,75%)	**<> L1+L3**		**<> L2**	
									**C<>T**	**CC<>CT+TT**	**C<>T**	**CC<>CT+TT**
C	2082 (74,41%)	1174 (74,78%)	320 (69,87%)	783 (70,54%)	1103 (70,34%)	339 (75,33%)	1442 (71,45%)	2616 (72,91%)	1.23; p = 0.00371	1.27; p = 0.00737	0.95; p = 0.67656	0.92; p = 0.54665
T	716 (25,59%)	396 (25,22%)	138 (30,13%)	327 (29,46%)	465 (29,66%)	111 (24,67%)	576 (28,54%)	972 (27,09%)	**Armitage's trend**		**Armitage's trend**	
									1.20; p = 0.00528		0.97; p = 0.68946	

C<>T allele frequency difference.

CC<>CT+TT allele positivity; frequent homo vs heterozygous and rare homozygous.

The different distribution of genotypes is demonstrated for each group and subgroup: controls, inflammatory bowel disease (IBD), Crohn's disease (CD), ulcerative colitis (UC), CD with solely colonic involvement (L2) and CD with solely ileal (L1) as well as ileal and colonic involvement (L3). Differences in genotype distribution compared to controls in general as well as the amount of all carriers (allele positivity) were subject to t- tests in patients with UC, CD and the CD subgroups L2 as well as L1+L3. Finally, results of the Armitage's trend tests for verification of significant associations of the rare T- variant are shown.

**Table 3:** Patients and controls sub grouped according to age and gender. Shown are percentages of individuals per group as well as the *TCF-4* (*TCF7L2*) rs3814570 T- allele frequency (minor allele frequency MAF). Differences in genotype distribution compared to controls in general as well as the amount of all carriers (allele positivity) and the amount of homozygous carriers were subject to t- tests in patients with ileal CD. Finally, results of the Armitage's trend tests for verification of significant associations of the rare T- variant are shown.

Age and gender										
overall		controls*	L1	L2	L3	ileal CD	CD		controls <> L1+L3	
Age groups	**A1** (<16 Y)	*2,42%*	5,88%	6,02%	9,76%	8,64%	8,06%	statistics for A3	**C<>T**	**CC<>CT+TT**
	MAF	*34,85%*	26,92%	23,08%	23,58%	24,24%	24,05%		1.37; p = 0.06312	1.32; p = 0.20576
	**A2**(16–40 y)	58,22%	72,85%	68,06%	80,85%	78,53%	76,22%		Armitage's trend	CC<>TT
	MAF	**25,28%**	31,06%	24,15%	29,61%	**30,00%**	28,85%		1.37; p = 0.07315	2.02; p = 0.04347
	**A3**(>40 y)	39,35%	21,27%	25,93%	9,39%	12,83%	15,71%	statistics for A2	**C<>T**	**CC<>CT+TT**
	MAF	**25,28%**	27,66%	23,21%	35,29%	**31,63%**	28,57%		1.27; p = 0.00567	1.36; p = 0.00438
gender	**male/M**	58,21%	50,88%	35,11%	40,43%	43,46%	41,59%		**Armitage's trend**	**CC<>TT**
	MAF	25,63%	33,91%	20,89%	28,57%	30,38%	28,59%		1.22; p = 0.00818	1.39; p = 0.08396
	**female/V**	41,79%	49,12%	64,89%	59,57%	56,54%	58,41%			
	MAF	25,44%	26,13%	26,71%	30,15%	29,14%	28,53%			

MAF = minor allele frequency.

* controls A1 only from Leuven.

### Sequencing of *TCF-4* promoter and gene region

To determine possible genetic variants in the *TCF-4* promoter, we sequenced the 2.1 kb upstream region of randomly selected healthy controls (n = 10) and patients with ileal CD (n = 10). In addition, we sequenced the region of the *TCF-4* gene in which functional insertions and deletions have been reported in colonic cancer [22]. Subsequently, a sequence analysis of known *TCF-4* exons was carried out, including ∼100 bp intron boundaries, to identify additional potential variants of this gene in these regions. Primers were designed using ENSG00000148737 of the Ensemble genome browser database for the promoter and exon sequencing. Sequencing was performed according to standard procedures and the primers are provided upon request.

### *TCF-4* genotyping

Leukocyte DNA was isolated by standard procedures (QIAamp DNA Blood Mini Kit, Qiagen, Hilden, Germany) from whole blood samples. Genotyping of the samples from the cohorts from Vienna and Leuven was performed using the matrix assisted laser desorption/ionization time-of-flight (MALDI-TOF) based mass spectrometry (MS) of allele specific primer extension products with a system from Bruker (Daltonik,Leipzig, Germany). Presence of *TCF-4* SNPs detected by MALDI-TOF MS was confirmed by TaqMan® analysis and direct sequencing in a subset of samples. MALDI-TOF MS based genotyping of the DNA samples obtained from Oxford was carried out using a MassARRAY® Compact System from Sequenom (San Diego, USA). Primers were designed using reference sequence NT 030059 and will be provided on request.

### *NOD2* genotype analysis

Genotyping for the common *NOD2* variants (SNP8, SNP12, and SNP13) was performed in the Vienna patient samples using TaqMan technology (Applied Biosystems, Foster City, California, USA), as described previously [7].

### Computer analysis and statistics

*In silico* screen of a 10 kb *TCF-4* upstream region was performed using “Promoter 2.0: for the recognition of PolII promoter sequences.” TESS (Transcription Element Search System) database software allowed assessing of potential binding sites for certain transcription factors in the candidate sequence. Polymorphisms were tested for Hardy–Weinberg equilibrium using Finetti specialized software (http://ihg2.helmholtz-muenchen.de/cgi-bin/hw/hwa1.pl) using log likelihood ratio chi square test in the three cohorts. For genetic analysis (comparing IBD subgroups versus controls) we used this software to calculate odds ratios, Confidence Intervals (C.I.) and to perform Pearson's goodness-of-fit chi-square tests. Differences in genotype frequencies were subject to both *t* tests and Armitage's trend tests. Values below 0.05 were considered significant. Linkage disequilibrium between *TCF-4* SNPs and haplotype blocks were calculated and identified using Haploview. To exclude a coincidental association of the SNP rs3814570, the significance of p- values<0.05 was verified using Benjamini- Hochberg correction in the overall group.

## Results

### SNP selection and haplotypes

**Figure 1:**
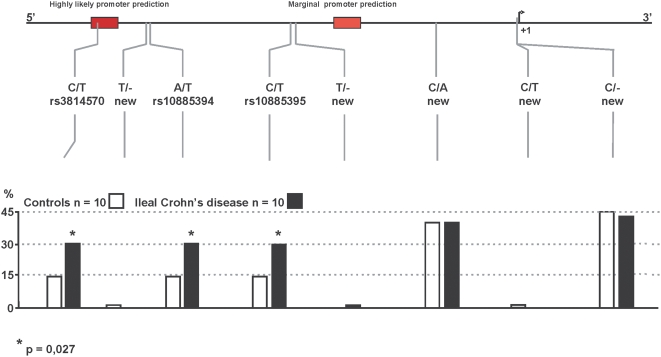
Sequencing of *TCF-4* (*TCF7L2*) 5′upstream putative promoter region. Sequencing of a 2.1 kb upstream region was performed in 10 healthy controls and 10 patients with ileal Crohn's disease. Putative regulatory regions were determined using promoter prediction software. Likely and marginal prediction sites are depicted as red boxes (upper panel). Relative location of identified variants is marked via grey dashes (upper part) and their allele frequency is demonstrated via bars for controls as well as patients (lower part).

**Figure 2:**
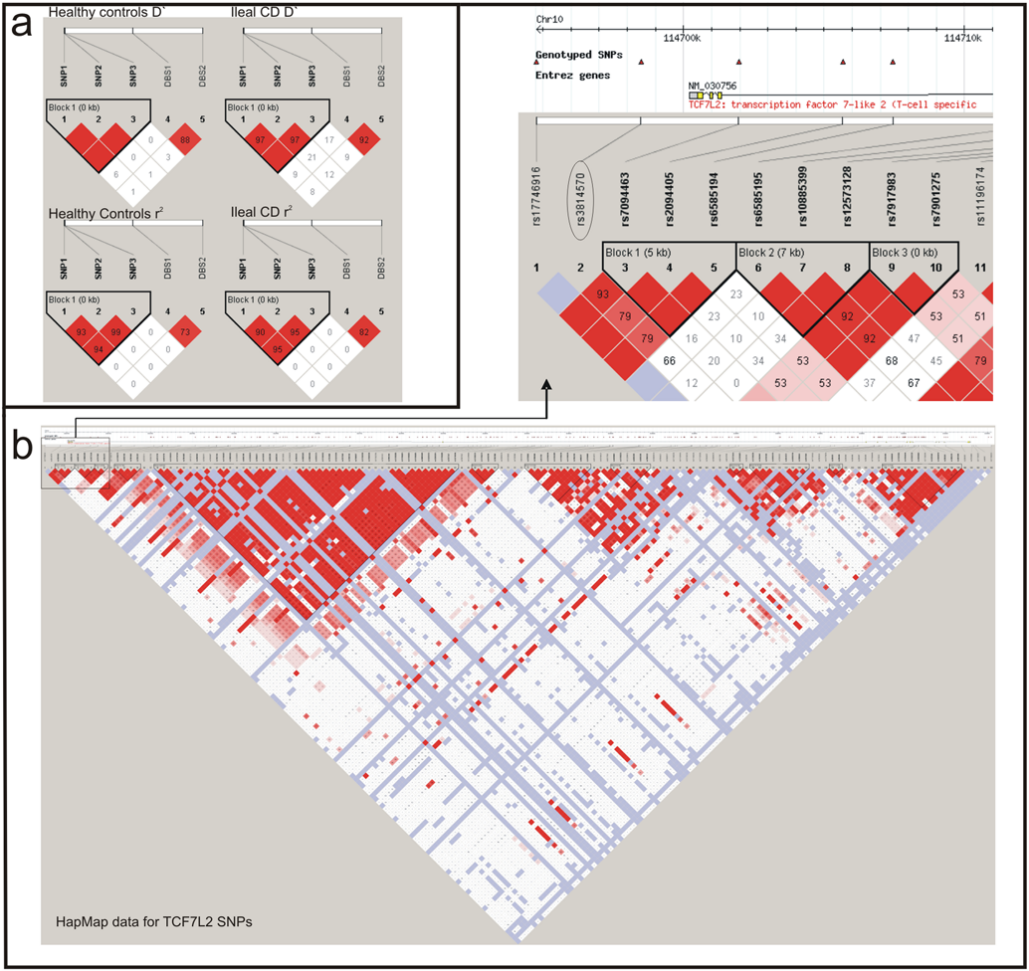
Distribution of haploblocks of *TCF-4* (*TCF7L2*). Both colour schemes (a and b) illustrate the linkage disequilibria. The variants are listed in the upper part of a and b, respectively. Haplotypes for *TCF-4* (*TCF7L2*) rs3814570 (SNP1), rs10885394 (SNP2), rs10885395 (SNP3) and SNPs associated with diabetes in the Vienna cohort are shown in a. A missing number for D′ or r^2^ equals 1. Figure 2b: HapMap data based haplotype blocks and linkage disequilibria (LD) for *TCF-4* (*TCF7L2*) polymorphisms. The intensity of red colouring in b is proportional to the extent of D′ or *r* ^2^ respectively and a missing number for each of them equals. The observed SNP in the putative promoter region is not part of any significant haplotype block.

To investigate potential genetic linkage of *TCF-4* to ileal CD, we screened for SNPs by sequencing 2.1 kb of the 5′ flanking region of *TCF-4* in a random group of 10 ileal CD patients and 10 healthy controls. We found eight SNPs in this putative promoter region (Figure 1), of which three (rs3814570, rs10885394, rs10885395) were in linkage disequilibrium (LD) in both the patient and control groups. In the control group, two of ten individuals carried the variants; in patients with ileal CD, six of ten individuals were heterozygous for the SNPs. On the basis of these findings, we studied a well-defined cohort of patients with CD and healthy controls from Vienna, Austria. In both the control and CD groups, we found LD between the 3 SNPs that defined a novel haplotype block (Figure 2a).

An *in silico* promoter and transcription factor binding-site analysis of the sequenced region revealed a potential regulatory region close to the location of rs3814570. Because of (i) the observed decreased expression of *TCF-4* mRNA, (ii) the higher frequency of the promoter variant in patients as well as (iii) the presence of a putative regulatory locus, we tested the hypothesis that rs3814570 exhibits an association with small intestinal involvement of CD. To exclude additional major variants in the gene region and possible LD of the identified promoter SNPs to other potentially functional variants in the *TCF-4* gene, we sequenced known coding exons, with ∼100 kb overlapping intron boundaries in 10 randomly chosen controls (6 identical to promoter analysis) as well as 25 patients with ileal CD (7 identical to promoter analysis) (Figure S1). We found ten additional putative SNPs, of which two were in LD, but none exhibited LD with the described promoter SNPs (data not shown). A further search for haplotypes in *TCF-4* was conducted based on published data from the HapMap project (Figure 2b), and no haplotype block including rs3814570 or additional SNPs in the gene region were identified.

### A *TCF-4* promoter variant is associated with ileal CD predisposition

**Figure 3:**
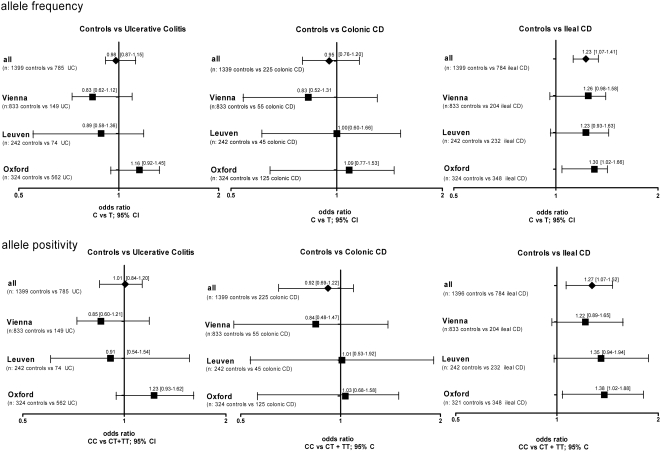
Differences in frequency distribution of rs3814570 in the different disease subgroups compared to healthy controls. Odds ratios and confidence intervals for the different comparisons are shown. The frequency distribution of rs3814570 was analyzed in different cohorts and combined samples: odds ratios and 95% confidence interval for allele frequency (upper panel) and allele positivity (amount of all T- allele carriers, lower panel) are shown for patients with ulcerative colitis (UC) (left panel), Crohn's disease (CD) patients with solely colonic involvement (L2, middle panel) and finally patients classified as either L1 (solely ileal) or L3 (ileal and colonic involvement) (right panel) compared to healthy control individuals.

Analysis of SNP rs3814570 frequency distribution was carried out in a total of 1399 controls (T allele frequency = 25.59%), 785 UC patients (T allele frequency = 25.22%), 225 CD patients with L2 classification (T allele frequency = 24.67%), and 784 CD patients with ileal involvement (L1+L3) (T allele frequency = 29.66%). In contrast to UC (OR 0.98, 95% CI 0.85 to 1.13, n.s.) which was similar to controls, the CD patients in aggregate exhibited a weak association for the minor variant (T allele positivity: OR 1.18, 95% CI 1.01 to1.39, p = 0.04358) (Table 2). Consistent with our initial hypothesis, investigation of the different CD subgroups revealed an association of the variant (T) with ileal CD (OR 1.23, 95% CI 1.07 to 1.41, p = 0.00371), but not with colonic CD (OR 0.95, 95% CI 0.76 to 1.20, ns). Testing for allele positivity by analyzing wildtype homozygous individuals (CC) versus all carriers of the minor variant (CT+TT), revealed the effect more clearly comparing healthy controls versus ileal CD (OR 1.27, 95% CI 1.07–1.52, p = 0.007372). Odds ratios and confidence intervals of the group analysis' in the respective cohorts as well as the combined analysis of all genotyping results are shown in Figure 3.

Since there were differences in allele frequencies between the cohorts (Tables S1, S2, and S3), we tested if those apparent frequency differences were statistically significant. In general the Oxford cohort exhibited a lower T allele frequency in controls (23.30%) compared to Leuven (26.65%) as well as to Vienna (26.17%) The same was true for CD patients (T allele frequency in Oxford: 27.38%, Leuven: 30.14% and Vienna: 28.96%), but could partly be explained by the different percentage of colonic CD patients in the groups. For CD with ileal involvement only, the frequency distributions in the cohorts were more similar (T allele frequency in Oxford: 28.30%, Leuven: 30.82% and Vienna: 30.64%) and not significantly different. Even though we found a possible change in frequency distribution between the Oxford control group with both the Leuven (allele frequency: OR 1.20, 95% CI 0.91 to 1.57, p = 0.19618) and Vienna controls (allele frequency: OR 1.17, 95% CI 0.94 to 1.44, p = 0.15453), the differences did not achieve statistical significance. The elevated SNP frequency in ileal CD patients was seen in three independent European cohorts, and a distinct significant association of the minor variant for rs3814570 with ileal CD could be observed in the combined analysis of all samples (Table 2).

### The association of rs3814570 with ileal CD is independent of gender but slightly more pronounced in patients >40 years

To make sure there is no disarrangement of age as well as gender we subgrouped all controls as well as the CD patient groups according to these criteria (Table 3). There were no consistent differences in allele frequency between men and women in either controls or patients; therefore we exclude a gender specific effect of the variant. Interestingly we found an increased association of the variant comparing patients with ileal, but not solely colonic CD of the age group A3 (>40 years) with controls of the same age group in the overall analysis, as well as in two separate cohorts (Leuven and Oxford). In the overall analysis a statistical significance for homozygous carriers was present (homozygous carriers: OR 2.02, 95% CI 1.01 to 4.05, p = 0.04347)

### rs3814570 shows the highest frequency in patients with stricturing ileal Cohn's disease

We grouped the patients according to their behaviour into B1 (inflammatory), B2 (stricturing) and B3 (penetrating) (Table 4). We found the highest frequency in the overall analysis within the ileal CD subgroup with stricturing behaviour (T allele frequency: 31.25%). This was also obvious in 2 separate cohorts (T allele frequency in Oxford: 29.81%, Leuven: 35.83%) but not seen in L2 CD patients. The association of the SNP with stricturing ileal CD compared to healthy controls exhibited a high significance in the overall analysis (allele frequency: OR 1.32, 95% CI 1.08 to1.62, p = 0.00686) and an additionally increased amount of homozygous carriers was observed (homozygous carriers: OR 1.71, 95% 1.11 to 2.63, p = 0.01460). To identify a possible association with aggressiveness of disease we also grouped the patients in such that have had at least one surgery for CD and those who did not (Table 4). No consistent result was observed; even though in two cohorts a trend towards a higher frequency in the ileal CD group with surgery (T allele frequency in Oxford: 28.93% and Leuven: 31.58%) and a significant stronger association with ileal CD in the surgery group compared to controls in one cohort (Oxford allele frequency: OR 1.34, 95% CI 1.03 to 1.74, p = 0.02885) was present.

**Table 4:** Patients sub grouped according to disease behaviour, L4 phenotype and surgery.

Disease behaviour and severity									
overall		L1	L2	L3	ileal CD	CD		controls <> L1+L3	
disease behaviour	**B1** (inflammatory)	17,12%	67,71%	22,20%	20,73%	31,31%			
	MAF	26,32%	24,17%	28,10%	27,67%	25,97%			
	**B2** (stricturing)	50,90%	7,17%	27,71%	34,42%	28,28%	statistics for stricturing behaviour	**C<>T**	**CC<>CT+TT**
	MAF	30,97%	21,88%	31,46%	**31,25%**	30,71%		1.32; p = 0.00686	1.34; p = 0.02745
	**B3** (penetrating)	31,98%	25,11%	50,09%	44,85%	40,40%		**Armitage's trend**	**CC<>TT**
	MAF	31,69%	26,79%	28,02%	28,78%	28,50%		1.30; p = 0.00963	1.71; p = 0.01460
upper GI involvement	**L4**	11,84%	8,00%	16,03%	14,80%	13,27%	statistics for L4	**C<>T**	**CC<>CT+TT**
	MAF	35,19%	22,22%	31,25%	**32,17%**	30,83%		1.38; p = 0.02882	1.50; p = 0.03749
	**no L4**	88,16%	92,00%	83,97%	85,20%	86,73%		**Armitage's trend**	**CC<>TT**
	MAF	29,60%	24,88%	29,18%	29,31%	28,25%		1.33; p = 0.03561	1.68; p = 0.10543
surgery	**at least one**	77,97%	33,33%	64,68%	68,54%	60,68%			
	MAF	30,79%	24,00%	29,11%	29,66%	28,97%			
	**no**	22,03%	66,67%	35,32%	31,46%	39,32%			
	MAF	29,00%	25,00%	29,59%	29,47%	27,78%			

MAF = minor allele frequency.

Shown are percentages of individuals per group as well as the *TCF-4* (*TCF7L2*) rs3814570 T- allele frequency (minor allele frequency MAF). Differences in genotype distribution compared to controls (all controls from Table 2) in general as well as the amount of all carriers (allele positivity) and the amount of homozygous carriers were subject to t- tests in patients with ileal CD. Finally, results of the Armitage's trend tests for verification of significant associations of the rare T- variant are shown.

### rs3814570 confers to the risk of an additional L4 phenotype in patients with ileal CD

To specifically address the question of upper GIT involvement (L4) we separated the patient groups in further subgroups according to this specific additional phenotype. In general the amount of patients with upper GIT involvement was quite low: Leuven patients with additional L4 phenotype: 12 patients L3; 4 patients L2; 6 patients L1; Oxford patients with additional L4 phenotype: 36 patients L3; 4 patients L2; 10 patients L1; Vienna patients with additional L4 phenotype: 40 patients L3; 10 patients L2; 11 patients L1. Comparing the allele frequencies with controls, we found a slight increase in patients with ileal CD and additional L4 phenotype (T allele frequency: 32.17%). This did not account for L2 patients with upper GIT involvement. The stronger association of the rare variant was also statistically significant in the overall analysis (allele frequency: OR 1.38, 95% CI 1 1.03 to1.84, p = 0.02882).

### rs3814570 is independent of *NOD2*

Given that the 3020insC frameshift mutation (SNP13) in *NOD2* is a known susceptibility factor for CD of the ileum and is associated with reduced HD-5 and-6 levels, we investigated if the observed association of rs3814570 with ileal CD is independent of *NOD2* in the Vienna and Leuven cohorts. We previously reported that the effects of reduced TCF-4 on Paneth cell α-defensins in ileal CD patients were independent of the effects of the SNP13 *NOD2* variant, since patients with this *NOD2* mutation showed a much more marked decrease of HD-5 and -6 expression [9]. The independence of the factors suggests that excluding patients harbouring *NOD2* SNP13 should yield similar allele frequencies of rs3814570 in the remaining ileal CD patients. Indeed, comparing all Leuven ileal CD patients (n = 232) to a subgroup excluding patients harbouring SNP13 (n = 191), there were no differences in allele frequency (OR 0.99) or allele positivity (OR 0.98). The same was true for the Vienna ileal CD patients (n = 204): following SNP13 exclusion (n = 154) the allele frequency gave an OR 1.06 and an allele positivity of OR 1.04. Thus, exclusion of patients with *NOD2* frameshift mutation SNP13 does not alter the observed allele frequencies of rs3814570 in patients with ileal CD, supporting independent effects of this *TCF-4* SNP and *NOD2* SNP13 in ileal CD.

## Discussion

In a hypothesis driven candidate gene approach, we investigated the association of sequence polymorphisms in the *TCF-4* (*TCF7L2*) promoter with ileal Crohn's disease. The reported findings represent the third identified genetic association with a link to Paneth cells in ileal CD. Recently Cadwell et al. published that Crohn's disease patients homozygous for the disease risk allele of *ATG16L1* display Paneth cell abnormalities which were also present in ATG16L1^HM^ mice [23]. Earlier we and others have shown that the 3020insC (SNP13) mutation in the intracellular, in Paneth cell present muramyl dipeptide receptor *NOD2*, is associated with especially reduced levels of HD5 and -6 [7], [8], [12]. Such a distinct deficiency in the innate defence also characterizes *NOD2* knock out mice [24]. The characteristic decrease of HD-5 and -6 in ileal CD results in an impaired innate immunity at the small intestinal barrier which is distinguished by reduced antibacterial activity in the epithelium, and proposed to disrupt the host - microbe balance at the mucosa [7], [8], [12]. It is also apparent in patients with wild type *NOD2* or either missense mutations (SNP8 and SNP12) but was, however, more pronounced in patients with the frameshift (SNP13) mutation. Different studies point to a delicate balance between commensal microbes and the intestinal mucosa (for review [25], [26]). We propose that a perturbation in this dynamic interplay has an important role in IBD pathogenesis [27]. In summary, *NOD2* SNP13 can in part explain a loss in HD-5 and -6 level but is found in only a minority of patients with ileal CD, but diminished defensin levels are present in the majority [8] and have an immediate effect on antimicrobial activity against and composition of the intestinal microflora [8]. A different functional link in ileal CD leading to diminished Paneth cell α-defensins HD-5 and -6, is a reduced mRNA expression of *TCF-4*, which has been previously reported by our group [9]. The identification of *TCF-4* as a new factor in the pathogenesis of ileal CD provides a more general mechanism for the deficit in HD-5 and -6. Since TCF-4 binds to and directly regulates the promoter regions of *HD-5* and *HD-6*, diminished *TCF-4* expression, maybe consequent on a genetic mutation, could account for a decrease in both of these defensins. The current data give support to the hypothesis of a genetic association between a rare SNP variant of *TCF-4* and ileal involvement in CD in a subset of patients. This variant, the rs3814570 T allele in the *TCF-4* promoter region, was most prevalent in CD localized to the ileum and no association with either colonic CD or UC was found. The strongest association with the variant was present in ileal CD patients with stricturing disease behaviour as well as those with an additional involvement of the upper GIT. The fact that genetic variants in TCF-4, a factor indispensable for Paneth cell function, are specifically associated with ileal CD provides further evidence that a decrease in HD-5 and -6 is predisposing and can be seen as a primary defect in the disease. The reported association between the *TCF-4* SNP and small intestinal involvement was found in all cohorts from Vienna, Oxford and Leuven. Variability of allele frequencies in controls between the Oxford cohort and the two cohorts from the European mainland might be explained by population differences as a consequence of a heterogenic ethnical history. For *NOD2* an even greater heterogeneity among Europeans has been reported [28]. In a letter regarding the frequency variability of DLG5 polymorphisms, another gene reported to exhibit an association with IBD, Tenesa et al. caution against pooling data from different populations, because true but in the cohorts different effects might be concealed [29]. The overall analysis of controls versus ileal CD showed a marginally lower difference when compared with the individual results of the Vienna and Oxford cohorts (Figure 3), so this might play a role in our analysis. However, differences in overall allele frequencies in the control cohorts were not statistically different.

Associations of genetic variations of the *TCF-4* gene with other diseases exist, but data are limited. An association of two non-coding SNPs in the *TCF-4* gene has been observed with diabetes mellitus [30] and, in another study, an association with deletions and insertions of adenines in the coding region was reported in patients with colorectal cancer [22]. We did not find any genetic association of these polymorphisms in UC nor CD (or in any of the clinical subgroups) in the samples from Vienna (Figure 2a and data not shown for repetitive A polymorphic region).

Given that Wnt/TCF-4 plays a major role in Paneth cell maturation, aside from its direct function in the expression of Paneth cell α-defensins [31], [32], the observed link between ileal CD and *TCF-4* suggests that impaired cell differentiation might be involved in the disorder. This would differ from many other views on IBD pathogenesis which emphasize the role of dysregulated immune function in otherwise normally functioning cells. If indeed a hypothesis on aberrant cell maturation proves significant, effective new therapeutic strategies might alternatively target steps in differentiation in addition to regulate or influence downstream impaired effector molecules like HD-5 and -6.

## Supporting Information

Figure S1 Sequencing of *TCF-4* (*TCF7L2*) exon regions and intron boundaries. Sequencing of exon regions was performed in a representative and limited number of healthy controls as well as Crohn's disease patients with known clinical phenotype (small intestinal CD). The relative location of identified variants is marked via grey dashes (upper part) and their allele frequency is demonstrated via bars for controls as well as patients (lower part). P<0,05 is considered statistical significant.
(10.07 MB DOC)

Table S1 *TCF-4* (*TCF7L2*) rs3814570 frequency distribution and statistical analysis of Oxford cohort samples. The different distribution of genotypes is demonstrated for each group and subgroup: controls, inflammatory bowel disease (IBD), Crohn's disease (CD), ulcerative colitis (UC), CD with solely colonic involvement (L2) and CD with solely ileal (L1), and ileo-colonic CD (L3). Differences in genotype distribution compared to controls as well as the number of carriers (allele positivity) were subject to t- tests. Results of the Armitage's trend tests for verification of significant associations with the minor T- variant are shown.
(0.05 MB DOC)

Table S2 *TCF-4* (*TCF7L2*) rs3814570 frequency distribution and statistical analysis of Vienna cohort samples. The different distribution of genotypes is shown for each group and subgroup: controls, inflammatory bowel disease (IBD), Crohn's disease (CD), ulcerative colitis (UC), CD with solely colonic involvement (L2), CD with solely ileal (L1) and ileo-colonic CD (L3). Differences in genotype distribution compared to controls as well as the number of carriers (allele positivity) were subject to t- tests as well as Armitage's trend test.
(0.05 MB DOC)

Table S3 *TCF-4* (*TCF7L2*) rs3814570 frequency distribution and statistical analysis of Leuven cohort samples. The different distribution of genotypes is demonstrated for each group and subgroup: controls, inflammatory bowel disease (IBD), Crohn's disease (CD), ulcerative colitis (UC), CD with solely colonic involvement (L2), CD with solely ileal (L1) and ileo-colonic CD (L3). Differences in genotype distribution compared to controls as well as the number of carriers (allele positivity) were subject to t- tests as well as Armitage's trend test.
(0.05 MB DOC)
