# Supplementary figures and images for: Genetic Variants of Wnt Transcription Factor TCF-4 (TCF7L2) Putative Promoter Region Are Associated with Small Intestinal Crohn's Disease
Source: PLoS One. 2009 Feb 16;4(2):e4496. doi: 10.1371/journal.pone.0004496 (PMC2637978; doi:10.1371/journal.pone.0004496)

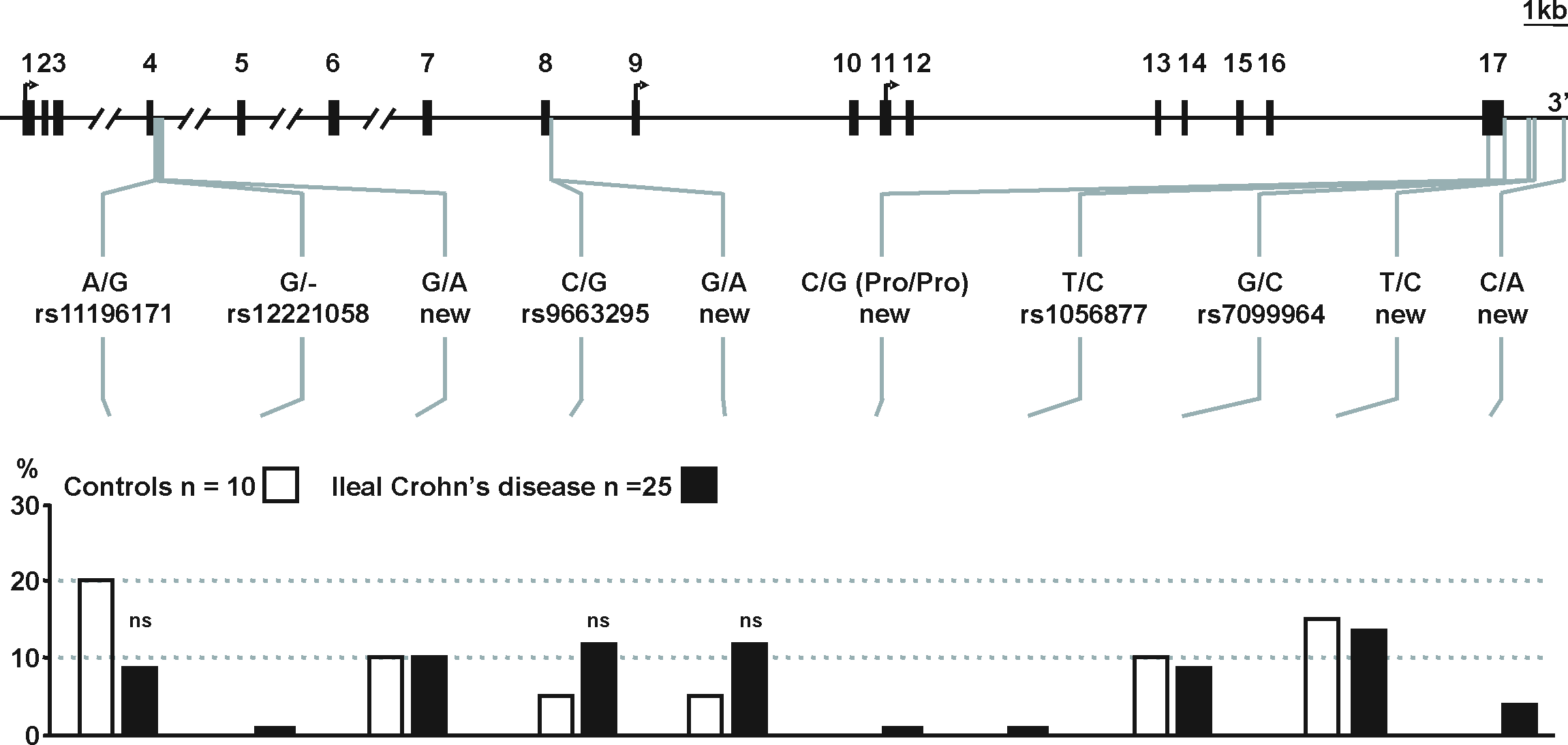

Supplement: Figure S1 — Sequencing of TCF-4 (TCF7L2) exon regions and intron boundaries. Sequencing of exon regions was performed in a representative and limited number of healthy controls as well as Crohn's disease patients with known clinical phenotype (small intestinal CD). The relative location of identified variants is marked via grey dashes (upper part) and their allele frequency is demonstrated via bars for controls as well as patients (lower part). P<0,05 is considered statistical significant. (10.07 MB DOC) [file pone.0004496.s001.doc]
