# Supplementary material for: Genetic Variants of Wnt Transcription Factor TCF-4 (TCF7L2) Putative Promoter Region Are Associated with Small Intestinal Crohn's Disease
Source: PLoS One. 2009 Feb 16;4(2):e4496. doi: 10.1371/journal.pone.0004496 (PMC2637978; doi:10.1371/journal.pone.0004496)
Supplement: Table S1 — TCF-4 (TCF7L2) rs3814570 frequency distribution and statistical analysis of Oxford cohort samples. The different distribution of genotypes is demonstrated for each group and subgroup: controls, inflammatory bowel disease (IBD), Crohn's disease (CD), ulcerative colitis (UC), CD with solely colonic involvement (L2) and CD with solely ileal (L1), and ileo-colonic CD (L3). Differences in genotype distribution compared to controls as well as the number of carriers (allele positivity) were subject to t- tests. Results of the Armitage's trend tests for verification of significant associations with the minor T- variant are shown. (0.05 MB DOC) [file pone.0004496.s002.doc]

Table S1

| **Oxford** | |  |  |  |  |  |  |  |  |  |  |  |
| --- | --- | --- | --- | --- | --- | --- | --- | --- | --- | --- | --- | --- |
|  | **controls** | **UC** | **CD (L1)** | **CD (L3)** | **CD (L1+L3)** | **CD (L2)** | **CD** | **IBD** | **controls** |  | **controls** |  |
|  | **n(%)** | **n(%)** | **n(%)** | **n(%)** | **n(%)** | **n(%)** | **n(%)** | **n(%)** | **<> CD** |  | **<> UC** |  |
|  |  |  |  |  |  |  |  |  | **C<>T** | **CC<>CT+TT** | **C<>T** | **CC<>CT+TT** |
| rs3814570 | 324 (100%) | 562 (100%) | 94(100%) | 254 (100%) | 348 (100%) | 125 (100%) | 473 (100%) | 1035 (100%) | 1.241; p=0.06745 | 1.282; p=0.09019 | 1.155; p=0.21020 | 1.225; p=0.15253 |
|  |  |  |  |  |  |  |  |  | **Armitage's trend** | | **Armitage's trend** | |
| C/C | 197 (60.80%) | 314 (55,87%) | 51 (54,26%) | 133 (52,36%) | 184 (52,87%) | 75 (60%) | 259 (54,76%) | 573 (55,36%) | 1.207; p=0.08218 |  | 1.117; p=0.22707 |  |
| C/T | 103 (31,79%) | 204 (36,3%) | 34 (36,17%) | 97 (38,19%) | 131 (37,64%) | 38 (30,4%) | 169 (35,73%) | 373 (36,04%) | **controls** |  | **controls** |  |
| T/T | 24(7,41%) | 44 (7,83%) | 9 (9,57%) | 24 (9,45%) | 33 (9,48%) | 12 (9,65) | 45 (9,51%) | 89 (8,6%) | **<> L1+L3** |  | **<> L2** |  |
|  |  |  |  |  |  |  |  |  | **C<>T** | **CC<>CT+TT** | **C<>T** | **CC<>CT+TT** |
| C | 497 (76,70%) | 832 (74,02%) | 136 (72,34%) | 363 (71,46%) | 499 (71,70%) | 188 (75,20%) | 687 (72,62%) | 1519 (73,38%) | 1.299; p=0.03645 | 1.383; p=0.03819 | 1.085; p=0.63631 | 1.034; p=0.87607 |
| T | 151 (23,30%) | 292 (25,98%) | 52 (27,66%) | 145 (28,54%) | 197 (28,30%) | 62 (24,80%) | 259 (27,38%) | 551 (26,62%) | **Armitage's trend** | | **Armitage's trend** | |
|  |  |  |  |  |  |  |  |  | 1.248; p=0.04537 |  | 1.102; p=0.65675 |  |
| C<>T | allele frequency difference | | |  |  |  |  |  |  |  |  |  |
| CC<>CT+TT | allele positivity; frequent homo vs heterozygous and rare homozygous | | | | | |  |  |  |  |  |  |
