# Supplementary material for: Genetic Variants of Wnt Transcription Factor TCF-4 (TCF7L2) Putative Promoter Region Are Associated with Small Intestinal Crohn's Disease
Source: PLoS One. 2009 Feb 16;4(2):e4496. doi: 10.1371/journal.pone.0004496 (PMC2637978; doi:10.1371/journal.pone.0004496)
Supplement: Table S2 — TCF-4 (TCF7L2) rs3814570 frequency distribution and statistical analysis of Vienna cohort samples. The different distribution of genotypes is shown for each group and subgroup: controls, inflammatory bowel disease (IBD), Crohn's disease (CD), ulcerative colitis (UC), CD with solely colonic involvement (L2), CD with solely ileal (L1) and ileo-colonic CD (L3). Differences in genotype distribution compared to controls as well as the number of carriers (allele positivity) were subject to t- tests as well as Armitage's trend test. (0.05 MB DOC) [file pone.0004496.s003.doc]

| **Vienna** | |  |  |  |  |  |  |  |  |  |  |  |
| --- | --- | --- | --- | --- | --- | --- | --- | --- | --- | --- | --- | --- |
|  | **controls** | **UC** | **CD (L1)** | **CD (L3)** | **CD (L1+L3)** | **CD (L2)** | **CD** | **IBD** | **controls** |  | **controls** |  |
|  | **n(%)** | **n(%)** | **n(%)** | **n(%)** | **n(%)** | **n(%)** | **n(%)** | **n(%)** | **<> CD** |  | **<> UC** |  |
|  |  |  |  |  |  |  |  |  | **C<>T** | **CC<>CT+TT** | **C<>T** | **CC<>CT+TT** |
| rs3814570 | 833 (100%) | 149 (100%) | 54 (100%) | 150 (100%) | 204 (100%) | 55 (100%) | 259 (100%) | 408 (100%) | 1.150; p=0.21117 | 1.125; p=0.40819 | 0.834; p=0.22244 | 0.852; p=0.37545 |
|  |  |  |  |  |  |  |  |  | **Armitage's trend** | | **Armitage's trend** | |
| C/C | 465 (55,82%) | 89 (59,73%) | 28 (51.85%) | 76 (50.67%) | 104 (50,98%) | 33 (60.00%) | 137 (52,90%) | 226 (55,39%) | 1.155; p=0.22915 |  | 0.824; p=0.23611 |  |
| C/T | 300 (36,01%) | 52 (34,9%) | 17 (31.48%) | 58 (38.67%) | 75 (36,76%) | 19 (34.55%) | 94 (36,29%) | 146 (35,78%) | **controls** |  | **controls** |  |
| T/T | 68 (8,16%) | 8 (5,37%) | 9 (16.67%) | 16 (10.67%) | 25 (12,25%) | 3 (5.45%) | 28 (10,81%) | 36 (8,82%) | **<> L1+L3** |  | **<> L2** |  |
|  |  |  |  |  |  |  |  |  | **C<>T** | **CC<>CT+TT** | **C<>T** | **CC<>CT+TT** |
| C | 1230 (73,83%) | 230 (77,18%) | 73 (67.59%) | 210 (70.00%) | 283 (69,36%) | 85 (77.27%) | 368 (71,04%) | 598 (73,28%) | 1.246; p=0.06870 | 1.215; p=0.21292 | 0.830; p=0.42498 | 0.842; p=0.54542 |
| T | 436 (26,17%) | 68 (22,82%) | 35 (32.41%) | 90 (30.00%) | 125 (30,64%) | 25 (22.73%) | 150 (28,96%) | 218 (26,72%) | **Armitage's trend** | | **Armitage's trend** | |
|  |  |  |  |  |  |  |  |  | 1248; p=0.08035 |  | 0.823; p=0.43956 |  |
| C<>T | allele frequency difference | | | |  |  |  |  |  |  |  |  |
| CC<>CT+TT | allele positivity; frequent homo vs heterozygous and rare homozygous | | | | | |  |  |  |  |  |  |

Table S2
