# Supplementary material for: Genetic Variants of Wnt Transcription Factor TCF-4 (TCF7L2) Putative Promoter Region Are Associated with Small Intestinal Crohn's Disease
Source: PLoS One. 2009 Feb 16;4(2):e4496. doi: 10.1371/journal.pone.0004496 (PMC2637978; doi:10.1371/journal.pone.0004496)
Supplement: Table S3 — TCF-4 (TCF7L2) rs3814570 frequency distribution and statistical analysis of Leuven cohort samples. The different distribution of genotypes is demonstrated for each group and subgroup: controls, inflammatory bowel disease (IBD), Crohn's disease (CD), ulcerative colitis (UC), CD with solely colonic involvement (L2), CD with solely ileal (L1) and ileo-colonic CD (L3). Differences in genotype distribution compared to controls as well as the number of carriers (allele positivity) were subject to t- tests as well as Armitage's trend test. (0.05 MB DOC) [file pone.0004496.s004.doc]

Table S3

| **Leuven** | |  | |  |  |  |  |  |  |  |  |  |  |
| --- | --- | --- | --- | --- | --- | --- | --- | --- | --- | --- | --- | --- | --- |
|  | **controls** | **UC** | | **CD (L1)** | **CD (L3)** | **CD (L1+L3)** | **CD (L2)** | **CD** | **IBD** | **controls** |  | **controls** |  |
|  | **n(%)** | **n(%)** | | **n(%)** | **n(%)** | **n(%)** | **n(%)** | **n(%)** | **n(%)** | **<> CD** |  | **<> UC** |  |
|  |  |  | |  |  |  |  |  |  | **C<>T** | **CC<>CT+TT** | **C<>T** | **CC<>CT+TT** |
| rs3814570 | 242 (100%) | 74 (100%) | | 81 (100%) | 151 (100%)* | 232 (100%) | 45 (100%) | 277 (100%) | 351 (100%) | 1.188; p=0.21390 | 1.289; p=0.14995 | 0.885; p=0.57247 | 0.910; p=0.72440 |
|  |  |  | |  |  |  |  |  |  | **Armitage's trend** | | **Armitage's trend** | |
| C/C | 135 (55.79%) | 43 (58,11%) | | 34 (41.98%) | 78 (51.66%) | 112 (48,28%) | 25 (55.56%) | 137 (49,46%) | 180 (51,28%) | 1.145; p=0.22827 |  | 0.877; p=0.58870 |  |
| C/T | 85 (35.12%) | 26 (35,145) | | 43 (53.09%) | 54 (35.76%) | 97 (41,81%) | 16 (35.56%) | 113 (40,79%) | 139 (39,6%) | **controls** |  | **controls** |  |
| T/T | 22 (9.09%) | 5 (6,76%) | | 4 (4.94%) | 19 (12.58%) | 23 (9,91%) | 4 (8.89%) | 27 (9,75%) | 32 (9,12%) | **<> L1+L3** |  | **<> L2** |  |
|  |  |  | |  |  |  |  |  |  | **C<>T** | **CC<>CT+TT** | **C<>T** | **CC<>CT+TT** |
| C | 355 (73.35%) | 112 (75,68%) | | 111 (68.52%) | 210 (69.54%) | 321 (69,18%) | 66 (73.33%) | 387 (69,86%) | 499 (71,08%) | 1.226; p=0.15631 | 1.352; p=0.10184 | 1.001; p=0.99783 | 1.009; p=0.97729 |
| T | 129 (26.65%) | 36 (24,32%) | | 51 (31.48%) | 92 (30.46%) | 143 (30,82%) | 24 (26.67%) | 167 (30,14%) | 203 (28,92%) | **Armitage's trend** | | **Armitage's trend** | |
|  |  |  | |  |  |  |  |  |  | 1.175; p=0.16888 |  | 0.997; p=0.99794 |  |
| C<>T | allele frequency difference | | | |  |  |  |  |  |  |  |  |  |
| CC<>CT+TT | allele positivity; frequent homo vs heterozygous and rare homozygous | | | | | | |  |  |  |  |  |  |
|  |  | |  |  |  |  |  |  |  |  |  |  |  |
